# Turpentine in Renal Hydatids

**Published:** 1905-09-16

**Authors:** 


### TURPENTINE IN RENAL HYDATIDS.

Dr. E. Mackey relates the case of a patient, a man aged 60, who for a period of 15 years had suffered from attacks of colic associated with the passage of hydatids in the urine. On examination a tumour of the size of a small orange was felt in the left kidney region, and the urine had a specific gravity of 1,034 and deposited urates and uric acid. He was ordered a mixture containing oil of turpentine, liquor potassæ, and mucilage. Subsequently he passed several hydatid vesicles but without any accompanying colic, and the renal tumour was appreciably smaller. The improvement has since been maintained and there has been no recurrence of the symptoms. Dr. Mackey quotes Dr. Warburton Begbie as favourable to the use of oil of turpentine in hydatids of the kidney.

